# The rationale for preventing cancer cachexia: targeting excessive fatty acid oxidation

**DOI:** 10.1186/s40880-016-0129-8

**Published:** 2016-07-21

**Authors:** Chao-Nan Qian

**Affiliations:** Department of Nasopharyngeal Carcinoma, Sun Yat-sen University Cancer Center; State Key Laboratory of Oncology in South China; Collaborative Innovation Center for Cancer Medicine, Guangzhou, Guangdong 510060 P.R. China

**Keywords:** Cachexia, Cancer, Fatty acid oxidation

## Abstract

Cachexia commonly occurs at the terminal stage of cancer and has largely unclear molecular mechanisms. A recent study published in *Nature Medicine*, entitled “Excessive fatty acid oxidation induces muscle atrophy in cancer cachexia,” reveals that cachectic cancer cells can secrete multiple cytokines that induce excessive fatty acid oxidation, which is responsible for muscle loss in cancer cachexia. Inhibition of fatty acid oxidation using etomoxir can increase muscle mass and body weight in cancer cachexia animal models. The usage of stable cachexia animal models is also discussed in this research highlight.

In the terminal stages of many chronic diseases, including cancer, loss of body mass may occur, which cannot be prevented or corrected by nutritional supplementation. This condition is termed cachexia, defined as >5% weight loss within 6 months. In addition to the loss of adipose tissue and overall metabolic imbalance, a typical characteristic of cachexia is muscle atrophy, resulting in fatigue and fetal weakness. Cancer cachexia is commonly observed in up to 80% of patients with advanced-stage disease and is one of the primary causes of cancer-related morbidity and mortality [1–3]. In fact, in cancer patients receiving radical therapy, cachexia can also occur. In patients with nasopharyngeal carcinoma from endemic areas, whose tumors are naturally sensitive to radiotherapy [4, 5], cachexia can be induced by radical radiotherapy in patients with post-irradiation nasopharyngeal necrosis [6]. Even worse, cachexia can usually induce resistance against conventional anti-cancer therapies, and no drug has been approved to treat or prevent cachexia in current medical practice [7].

It is believed that early diagnosis of and intervention in cancer cachexia can control its fetal progression, improve a patient’s quality of life, and prolong survival [8]. However, the underlying molecular mechanisms of cancer cachexia are largely unclear, preventing the development of effective intervention approaches. In a study recently published in *Nature Medicine*, entitled “Excessive fatty acid oxidation induces muscle atrophy in cancer cachexia,” Fukawa et al. [9] report their interesting findings that cachectic cancer cells secrete multiple inflammatory factors, including interleukin-6 and tumor necrosis factor-alpha, which have been suspected to play roles in cancer cachexia for decades [10, 11]. This secretion results in fatty acid oxidation and activation of a p38 stress-response signature in the skeletal muscles before presentation of cachectic muscle atrophy.

In this study, the authors also demonstrate that blockade of fatty acid oxidation using etomoxir can rescue human myotubes in vitro and can increase muscle mass and body weight in cancer cachexia animal models [9]. Therefore, targeting fatty acid-induced oxidative stress has a great potential for preventing cancer-induced cachexia.

The application of stable cachexia animal models is one of the strengths of this study. As shown in Fig. 1, the human clear cell renal cell carcinoma cell line RXF393 can stably induce cachexia in nude mice after several weeks of subcutaneous inoculation or orthotopic inoculation of the cancer cells into the subrenal capsule area.

**Fig. 1:**
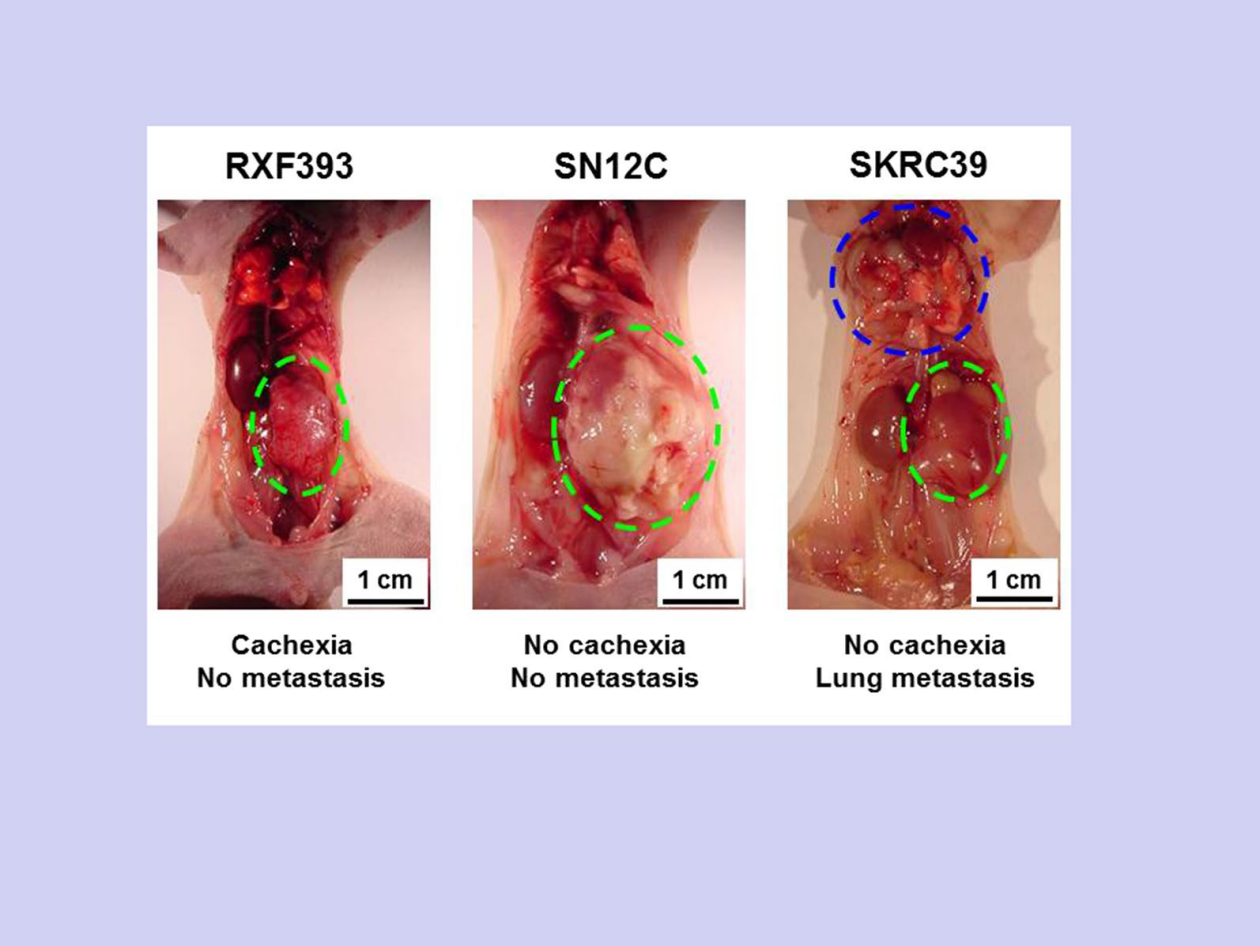
Different clear-cell renal cell carcinoma (ccRCC) cell lines have different biological behaviors after orthotopic inoculation of the cancer cells into the subrenal capsule area. Inoculation of cancer cells into the subrenal capsule area of nude mice to generate an orthotopic renal cancer model was applied to evaluate the different biological behaviors of ccRCC cells. The tumor composed of the RXF393 cell line induced cachexia in the host, leading to a moribund condition, even when the primary tumor was small (*green dashed circle*). The tumor composed of the SN12C cell line could not induce cachexia or metastasis, even when the primary tumor was very large (*green dashed circle*). The tumor composed of the SKRC39 cell line could generate heavy lung metastases (*blue dashed line*), but not cachexia

Much more information can be found in the authors’ dataset from high-throughput expression profiling of human myotubes after exposure to cachectic or non-cachectic conditioned media [9]. The involvement of multiple cytokines reported in this comprehensive study also indicates that certain other causal factors may play a role in cancer cachexia, and especially those factors responsible for adipose tissue rearrangement.
